# Regression of biventricular hypertrophy in acromegalic cardiomyopathy following management of excessive growth hormone secretion

**DOI:** 10.1093/omcr/omae112

**Published:** 2024-10-10

**Authors:** Raid Faraj, Thierno Hamidou Diallo, Mehdi Abdelali, Reda Lahjouji, Fatima-azzahra Benmessaoud, Nawal Doghmi, Jamila Zarzur, Mohamed Cherti

**Affiliations:** Cardiology B Department, Ibn Sina University Hospital, Mohammed V University of Rabat, Angle avenue Allal El Fassi et Mfadel Cherkaoui, Al Irfane - 8007, N.U, Morocco; Cardiology B Department, Ibn Sina University Hospital, Mohammed V University of Rabat, Angle avenue Allal El Fassi et Mfadel Cherkaoui, Al Irfane - 8007, N.U, Morocco; Cardiology B Department, Ibn Sina University Hospital, Mohammed V University of Rabat, Angle avenue Allal El Fassi et Mfadel Cherkaoui, Al Irfane - 8007, N.U, Morocco; Cardiology B Department, Ibn Sina University Hospital, Mohammed V University of Rabat, Angle avenue Allal El Fassi et Mfadel Cherkaoui, Al Irfane - 8007, N.U, Morocco; Cardiology B Department, Ibn Sina University Hospital, Mohammed V University of Rabat, Angle avenue Allal El Fassi et Mfadel Cherkaoui, Al Irfane - 8007, N.U, Morocco; Cardiology B Department, Ibn Sina University Hospital, Mohammed V University of Rabat, Angle avenue Allal El Fassi et Mfadel Cherkaoui, Al Irfane - 8007, N.U, Morocco; Cardiology B Department, Ibn Sina University Hospital, Mohammed V University of Rabat, Angle avenue Allal El Fassi et Mfadel Cherkaoui, Al Irfane - 8007, N.U, Morocco; Cardiology B Department, Ibn Sina University Hospital, Mohammed V University of Rabat, Angle avenue Allal El Fassi et Mfadel Cherkaoui, Al Irfane - 8007, N.U, Morocco

**Keywords:** acromegaly, biventricular hypertrophy, acromegalic cardiomyopathy, diastolic dysfunction, cardiovascular disease, growth hormone, case report

## Abstract

Acromegalic cardiomyopathy is a significant cardiovascular complication associated with acromegaly, caused by excessive growth hormone production from a pituitary adenoma. Early diagnosis can be challenging due to its insidious nature. This case underscores the critical significance of timely medical intervention, illustrating favorable outcomes resulting from prompt therapeutic measures.

## Introduction

Acromegaly is a rare and insidious condition primarily caused by a pituitary adenoma that results in excessive growth hormone (GH) production, leading to elevated levels of insulin-like growth factor 1 (IGF-1). This hormonal imbalance leads to abnormal growth of bones and soft tissues and disruption of carbohydrate metabolism, increasing the risk of cardiovascular pathologies [1]. Despite the presence of characteristic physical changes in acromegaly, early diagnosis is often challenging due to its insidious nature, resulting in a lack of awareness from the patient, their family, and even their healthcare provider. Consequently, multiple comorbidities are frequently present at the time of diagnosis [1, 2]. Although acromegaly is a rare condition, the associated comorbidities and long-term management impose a significant burden. Cardiovascular disease is the most prevalent comorbidity, accounting for up to 80% of complications [3]. The presence of cardiovascular comorbidities substantially increases the risk of morbidity and mortality, with an up to 50% increase in overall mortality, especially when combined with other comorbidities such as glucose intolerance or diabetes, dyslipidemia, and sleep apnea [4, 5]. While hypertension is the most commonly observed cardiovascular comorbidity, with a prevalence ranging from 18 to 60% [4], acromegalic cardiomyopathy has also been reported independently of hypertension [6]. This condition encompasses hypertrophic cardiomyopathy with progressive diastolic and systolic dysfunction, valvular anomalies, and arrhythmias [4]. While numerous publications have documented cases of acromegalic cardiomyopathy occurring during the progression of acromegaly, only a limited number have reported it as an initial manifestation of the condition and a complete regression of cardiac hypertrophy after the excessive GH secretion.

Our paper was written according to the CARE guidelines [7].

## Case presentation

**Figure 1:**
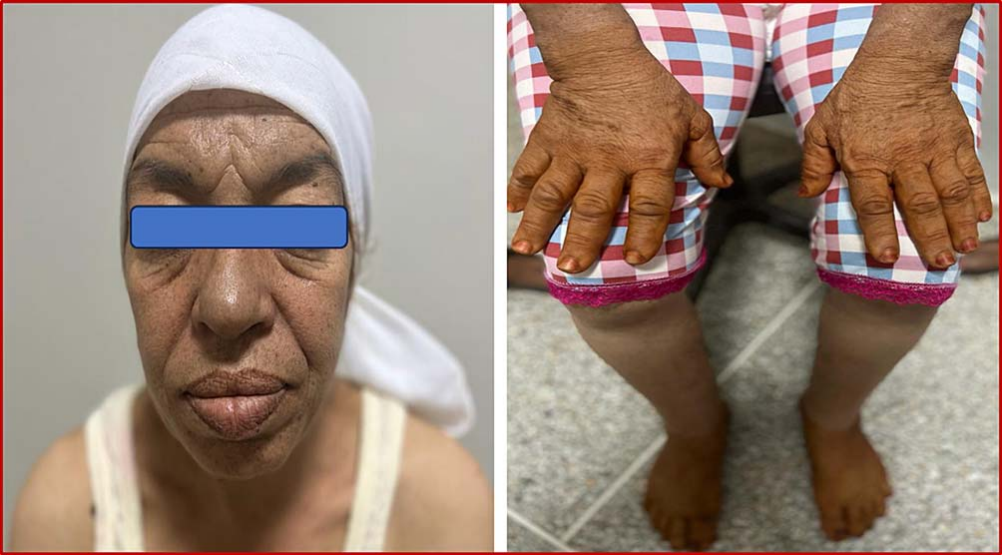
Physical examination findings. (**A**): macrognathia (Yellow half-circle), prominent and enlarged facial features affecting the facial bones, lips and nose (blue arrows), thickened skin on the forehead (green arrow), (**B**): enlargement of the hands and feet.

**Figure 2:**
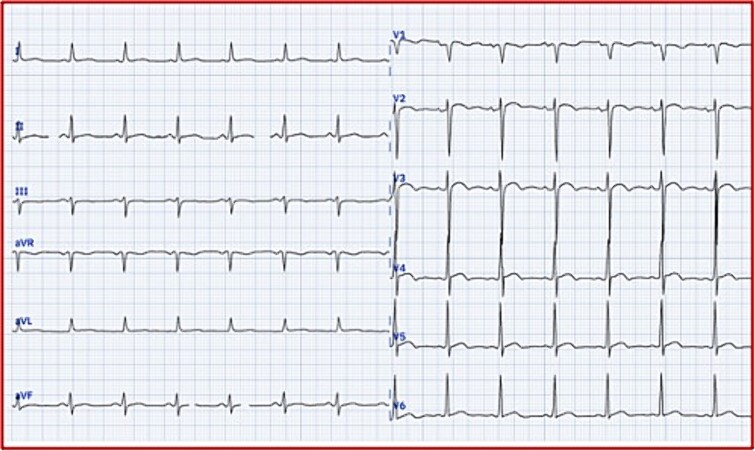
ECG findings. Electrical left ventricular hypertrophy based on the Cornell index (Sv3 + Ravl >20 mm).

**Figure 3:**
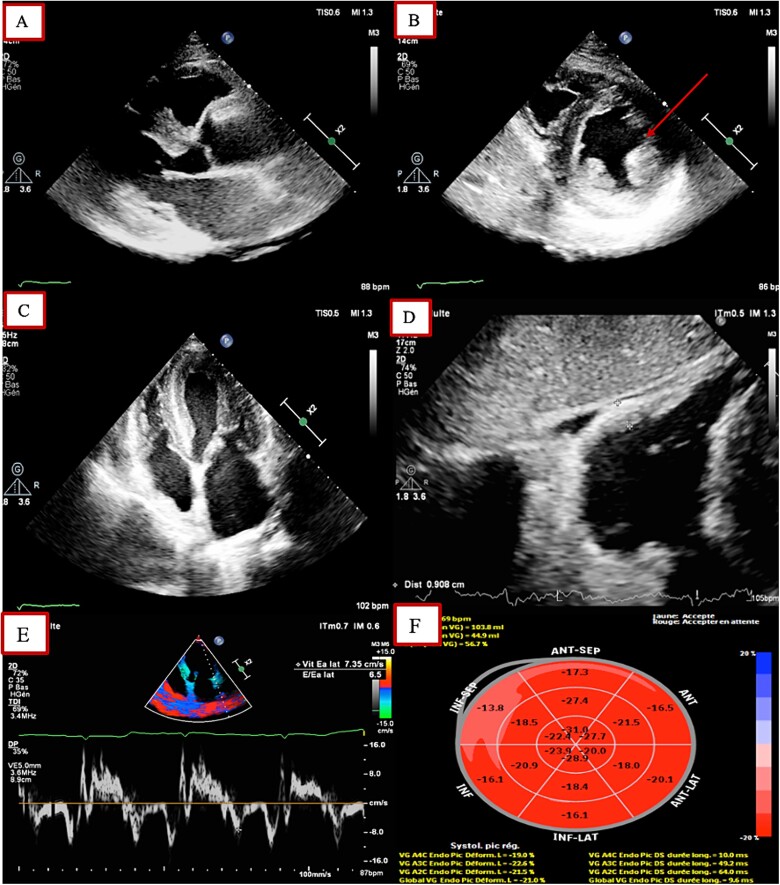
TTE findings. Parasternal long and short axis view showing concentric left ventricular hypertrophy with extension to the papillary muscles (red arrow). (**C**): 4 chamber view showing biventricular hypertrophy with left atrial enlargement (**D**): Subcostal view showing right ventricular hypertrophy. (**E**): Lateral E’ velocity suggesting diastolic dysfunction. (**F**): Normal global longitudinal strain calculated at −21%.

**Figure 4:**
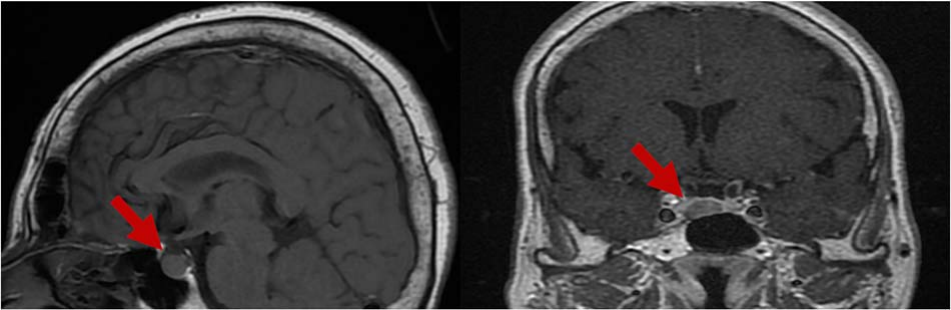
Pituitary MRI. Identification of of a somatotroph adenoma (red arrows).

A 52-year-old woman, devoid of identified cardiovascular risk factors and without a prior medical or surgical history, presented to our department with progressive dyspnea, fatigue, and weakness, significantly impacting her quality of life. Her blood pressure, heart rate, and respiratory parameters were within normal limits. We observed a diastolic murmur (graded 2/6), indicative of aortic regurgitation. Additionally, the patient exhibited facial and jaw enlargement with prognathism, prominent features affecting the lips, nose, and tongue, along with thickened skin (Fig. 1). Marked enlargement of the hands and feet was also noted. The ECG showed electrical left ventricular hypertrophy based on the Cornell index (Sv3 + Ravl >20 mm) (Fig. 2). The TTE results indicated the presence of biventricular hypertrophy with an elevated indexed left ventricular mass. The hypertrophy exhibited a concentric pattern with evidence of diastolic dysfunction based on lateral E’ Velocity and LAVI (left atrial volume index). The LV showed hyperkinesis, with an estimated ejection fraction of 70%. The GLS was within normal limits, calculated at −21%. Additionally, grade I aortic insufficiency was detected (Fig. 3, Video 1). Her BNP levels were notably elevated (400 pg/ml), which, in conjunction with her symptoms and the TTE findings, strongly suggested a high probability of HFpEF according to HFA-PEFF score. After excluding the most evident causes of LVH, such as hypertension, aortic stenosis, cardiac amyloidosis, and hypertrophic cardiomyopathy, our approach, guided by the patient’s typical signs of excess GH, commenced with evaluating pituitary function. The results indicated, as expected, elevated levels of IGF-1 up to 550 ng/ml (normal range typically between 80–280 ng/ml), which is acknowledged as one of the most reliable tests for diagnosing acromegaly. Other pituitary hormone levels were normal. Subsequent pituitary MRI confirmed the presence of adenoma (Fig. 4). Consequently, the diagnosis involved acromegaly complicated by HFpEF, a condition commonly referred to as acromegalic cardiomyopathy. Additionally, diastolic dysfunction was managed with a treatment regimen for HFpEF, incorporating SGLT2 inhibitors (Empagliflozin 10 mg/day) and diuretics (Furosemide 40 mg three times daily). The post-operative MRI, performed after 3 months, did not show any residual tumor. The patient’s clinical condition improved significantly, and she was no longer experiencing dyspnea. Furthermore, BNP levels, measured at 70 pg/ml, and IGF-1 levels, at 100 ng/ml, normalized within six months. Notably, the regulation of GH levels demonstrated a consequential reduction in left ventricular hypertrophy after nine months (Fig. 5—Video 2). Therefore, only SGLT2 inhibitors were maintained.

**Figure 5:**
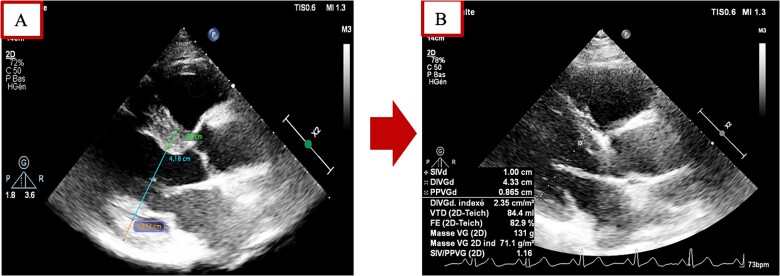
TTE performed before (**A**) and nine months after (**B**) showing a reduction in left ventricular hypertrophy following successful control of excessive growth hormone secretion and HFpEF treatment.

## Discussion

Acromegaly is a rare condition characterized by morphological abnormalities and systemic manifestations resulting from chronic exposure to growth hormone. Its total prevalence ranges from 28 to 137 cases per million. Cardiovascular disease is a common complication of acromegaly, accounting for approximately 60% of acromegaly-related mortality. Acromegalic cardiomyopathy, characterized by concentric ventricular hypertrophy, diastolic dysfunction, and progressive impairment of systolic function, follows hypertension as the second most frequent cardiovascular complication [4, 8].

Clinically, these alterations progress through three stages. The initial stage is the hyperkinetic syndrome, characterized by a hyperkinetic left ventricle with increased contractility, cardiac output, and low peripheral resistance. Concentric biventricular hypertrophy is present in over two-thirds of patients at this stage, but filling disorders are not yet observed [8]. The second stage is marked by the progression of hypertrophy, development of interstitial fibrosis, and impaired diastolic function, manifested by decreased E-wave filling, early to late mitral and tricuspid velocity ratio, and increased isovolumetric relaxation time. Most patients are diagnosed during this stage. The third stage is characterized by impaired systolic and diastolic function, ultimately leading to heart failure [4, 8]. In our patient, increased contractility with grade II diastolic dysfunction was observed, suggesting early cardiac involvement.

Age, arterial hypertension, disease activity, and duration have been identified as potential risk factors for left ventricular hypertrophy. However, the role of hypertension in the occurrence of acromegalic cardiomyopathy is still debated, as some studies found no significant difference in the prevalence of left ventricular hypertrophy between patients with and without hypertension [9]. Some authors suggest that hypertension may act as an aggravating factor in patients with acromegalic cardiomyopathy [4, 6, 8]. This is precisely the case with our patient, who exhibited ventricular hypertrophy despite the absence of hypertension.

Therapeutic modalities for acromegaly aim to normalize serum GH and IGF-1 levels, reduce mortality risk, improve clinical symptoms, and control tumor size through surgical or medical interventions [3, 8]. Successful surgical or pharmacological treatment has been shown to significantly improve prognosis, reduce left ventricular hypertrophy, and improve left ventricular filling. Transsphenoidal resection, the surgical removal of a pituitary adenoma, is the most cost-effective and efficient treatment for most cases of acromegaly.

Acromegaly is associated with a wide range of comorbidities, resulting in increased mortality rates compared to healthy individuals and a reduced average life expectancy of about 10 years [3]. Cardiovascular pathologies are the main risk factors for morbidity and mortality in acromegalic patients. However, early diagnosis and management, particularly before the age of 40, have been shown to reduce cardiovascular risk [10]. Fortunately, our patient was diagnosed early, allowing for prompt intervention and permitting a change in the trajectory of this disease long before it reaches the stage of complications.

## Novel teaching points

- To be able to diagnose acromegalic cardiomyopathy earlier and implement timely interventions to reduce cardiovascular risk.
- To understand the importance of regulating growth hormone levels in acromegaly patients with cardiac manifestations, recognizing its pivotal role in reducing mortality rates.

## Conclusion

Acromegaly is a complex disorder marked by significant cardiovascular comorbidities, contributing to heightened morbidity and mortality rates. This underscores the vital importance of detecting cardiac abnormalities in acromegaly and emphasizes the necessity for regulating hormone levels to achieve a substantial reduction in mortality rates.

## Supplementary Material

Clinical_significance_omae112

Video_1_omae112

Video_2_omae112

Video_caption_omae112

## Data Availability

Data sharing is not applicable to this article as no datasets were generated or analyzed during the current study.
