# Supplementary material for: Regression of biventricular hypertrophy in acromegalic cardiomyopathy following management of excessive growth hormone secretion
Source: Oxf Med Case Reports. 2024 Oct 10;2024(10):omae112. doi: 10.1093/omcr/omae112 (PMC11465510; doi:10.1093/omcr/omae112)
Supplement: Clinical_significance_omae112 [file clinical_significance_omae112.docx]

**Clinical significance :**

1. Cardiovascular comorbidities are frequent and paramount in acromegalic patients
2. They are the leading cause of morbidity and mortality in patients with acromegaly
3. The importance of early recognition and treatment long before reaching the stage of complications
