# Supplementary material for: Regression of biventricular hypertrophy in acromegalic cardiomyopathy following management of excessive growth hormone secretion
Source: Oxf Med Case Reports. 2024 Oct 10;2024(10):omae112. doi: 10.1093/omcr/omae112 (PMC11465510; doi:10.1093/omcr/omae112)
Supplement: Video_caption_omae112 [file video_caption_omae112.docx]

**Video Legends :**

**Video 1: [TTE,** before treatment**]**

**Video 2: [TTE,** nine months after treatment**]**
